# Occupational Noise on Floating Storage and Offloading Vessels (FSO)

**DOI:** 10.3390/s21051898

**Published:** 2021-03-08

**Authors:** Grzegorz Rutkowski, Jarosław Korzeb

**Affiliations:** 1 Department of Navigation, Faculty of Navigation, Gdynia Maritime University, 81-345 Gdynia, Poland; kptgrzegorzrutkowski@gmail.com; 2 Department of Fundamentals of Construction of Transport Equipment, Faculty of Transport, Warsaw University of Technology, 00-662 Warszawa, Poland

**Keywords:** occupational noise, marine transport, sector offshore, floating storage and offloading vessel FSO

## Abstract

The purpose and scope of this paper are to provide guidance of the potential impacts of being subjected to high level noise recorded on 1st generation (30 years old) floating storage and offloading vessels (FSO) in sector offshore. The international community recognizes that vibroacoustic impacts from commercial ships may have negative consequences for both humans (worker’s) and marine life, especially marine mammals. As regards the effect of noise on human health, there are legal requirements imposing the noise exposure control on personnel working on ships. The acceptable noise exposure standards are established in European Union Directive 2003/10/EC (2003), the NOPSEMA Regulation (2006), the Maritime Labor Convention (MLC) guidelines (2006), and the recommendations of the International Maritime Organization IMO contained, e.g., IMO MEPC.1/Circ.833 (2014). These regulations inform employers and employees what they must do to effectively protect both the marine environment and the health and life safety of workers employed in the maritime industry offshore. This study also presents an analysis of the results of noise measurements carried out on exemplary 1st generation FSO units.

## 1. Introduction

Noise is defined as unwanted sound that may damage a person’s hearing. The amount of damage caused by noise depends on the total amount received over time [1,2]. The degree of risk is affected by the intensity (loudness) and the frequency (pitch) of the noise, as well as the duration and pattern of exposure and the individual’s susceptibility to hearing impairment. Acceptable noise levels on ships are widely discussed in international standards, directives, conventions, guidelines, recommendations of marine operators [3,4,5,6].

The damage can be divided into short-term and long-term effects in non-auditory effects (can also be associated with mild exposure levels) and audio effects [1,7]. The most prominent effect among the auditory ones is the noise-induced hearing loss (NIPTS noise induced permanent threshold shifts). At first approximation the NIPTS are related to the noise total cumulated doses (a permanent threshold shift practically does not occur if the noise levels are lower than 80 dB(A)). The auditory effects are quantified by dose-effect relations. The algorithms for calculating the risk of noise-induced hearing loss are contained in the technical international standard ISO 1999. The noise exposure limits and the two action levels are based on the ISO 1999 risk curves (the action levels and the noise exposure limit value). This last is set at 87 dB(A) by the European Directive while if the second action level of 85 dB(A) is exceeded, the employer have to impose the use of hearing protection devices. The international community recognizes that noise from commercial ships may have both short and long-term negative consequences for both human (worker’s) life and marine life—especially marine mammals susceptible to underwater-radiated noise from ship’s sonars, propellers, and thrusters [8,9].

When efforts have been made to mitigate noise, as far as reasonable and practical, evaluation should be undertaken to determine the success or reduction efforts. The successful strategy to reduce radiated noise should consider interactions and contributions from measures provided to achieve other objectives such as reduction of onboard noise and improvements in energy efficiency [10,11]. According to MLC 2006 (Maritime Labour Convention from 2006) implemented for all shipping industry on 20 August 2013 (ratification date), accommodation, recreational, and catering facilities on ships shall meet the requirements in Regulation 4.3, and the related provisions in the Code, on health and safety protection and accident prevention, with respect to preventing the risk of exposure to hazardous levels of noise and other ambient factors. Acoustic insulation or other appropriate sound-absorbing materials should be used in the construction and finishing of bulkheads, deck heads, and decks within the sound-producing spaces as well as self-closing noise-isolating doors for machinery spaces. IMO also generated some guidelines for the reduction of underwater noise from commercial shipping to address the adverse impacts on marine life (e.g., IMO Resolution MEPC.1/Circ.833 (2014)).

The Control of Noise at Work Regulations (Noise Regulations) 2005 based on European Union Directive 2003/10/EC (2003) tell employers and employees what they must do to protect the hearing of those employed at work [4,5,12]. The Noise Regulations apply to all work activities on offshore installations, wells, pipelines, and pipelines works and to connected activities within the territorial waters of Great Britain or in the designated areas of the UK Continental Shelf. Similar requirements (e.g., specified in NOPSEMA Regulations (2006)) have been implemented for sector offshore in Australia and some others sectors offshore all around the world.

In fact, all ships owners are committed to aim to minimize the generation and emission of noise that lie within the scope of ALARP (as low as reasonably practical), and to set goals for peak and daily noise exposure levels at work [13,14,15,16]. Seafarers usually monitor noise exposure by recording noise in the daily/monthly noise log to identify and, where possible, correct high noise trends. In offshore industry on floating storage and offshore loading units (FSO) the audiometric testing equipment (which must comply with the noise standards as per the National Code of Practice) is always available to all crew members. The familiarization process for new crew members on the FSO includes the formal introduction of the Noise Control Policy and the Noise Management Plan. Each FSO has also its own ship-specific Noise Management Plan. Contractors are to comply with the noise standards as per the National Code of Practice.

The unit of sound level and noise exposure measurement is the Decibel (dB) and is expressed on a logarithmic scale. When considering the effect on human hearing, the A-weighted decibel dB(A) unit is used [17,18]. This takes account of the response of the ear to different frequencies. The noise measurement scale is logarithmic. In this scale, an increase of 3 dB(A) means doubling the energy. There is a simple guide to indicate whether there may be noise levels with potential for causing hearing damage. If you need to shout to be heard by someone about 2 m away the probable noise level is likely to be 85 dB. On all FSOs employers must prevent risks to their workers from exposure to excessive noise [19]. “Excessive noise” as specified in European Union Directive 2003/10/EC (2003) as well as in NOPSEMA Regulations (2006) means a level of noise above 85 dB(A) averaged over 8 hours’ period for noise exposure referenced to 20 micro Pascal’s (LAeq,8h); or LCpeak of 140 dB(C)—that is, a C-weighted peak sound pressure level of 140 dB(C) referenced to 20 micro pascals. 

## 2. Short-Term and Long-Term Effects of Exposure

The short-term effects from noise are stress, loss of sleep, temporary deafness, poor communication. All of the above can lead in human life to potential situations, which may result in accidents and incidents. The long-term effect from noise is deafness and connected disability is also possible.

Noise exposure exceeding LAeq,8h of 85 dB(A) presents a high risk to a person’s health and safety at the workplace. A person working with or near noisy equipment or processes may be affected by high direct or ambient noise and may develop noise-induced hearing loss in situations where no control measures have been put in place. Regular exposure to high noise levels causes, in time, hearing loss through the destruction of the delicate hair cells in the inner ear’s cochlea [20].

This is often accompanied by tinnitus, or ringing in the ears. Long-term occupational exposure to noise can negatively affect the behavior of the tympanic membrane (eardrum) and ossicles mechanical system, the functioning of the organ of Corti, as well as the cooperation in transmitting information to the Cochlear nerve and further to cerebral cortex [21,22,23]. The effect of the accommodation of the hearing organ caused by prolonged exposure may cause a permanent increase in the hearing threshold (Manson curves) or a selective increase in the hearing threshold for selected frequency bands, which results in speech incomprehensibility [24]. Health effects of noise exposure include: temporary threshold shift—occurs immediately after exposure to high noise levels (the condition may last for minutes to hours); noise induced hearing loss—occurs from long term exposure to high noise levels and is irreversible; tinnitus—ringing in the ears which sometimes accompanies noise induced hearing loss; and/or acoustic trauma—results from explosions or extremely loud impulses which may destroy hair cells and ear architecture [25].

Other effects from exposure to noise include increased heart rate and blood pressure, headache, irritability, nausea, insomnia, reduced concentration, and depression. In addition to the risk of temporary or permanent hearing loss, high noise levels may cause difficulties in verbal communication and in hearing warning signals or emergency commands [20,21,24].

As we can read in many publications [11,17,18]: below 35 dB (A)—noise is not harmful, can be very annoying or interfere with work, between 35 and 70 dB (A)—noise negatively affects the nervous system, leads to fatigue, decreased work efficiency, reduced speech intelligibility, between 70 and 85 dB (A)—noise significantly reduces work efficiency, can cause headaches and permanent hearing impairment, between 85 and 130 dB (A)—noise causes numerous damage to hearing, nervous system disorders, prevents speech intelligibility, between 130–150 dB (A)—noise causes permanent damage to hearing, and over 150 dB (A)–after about 5 min, noise paralyzes the functioning of the body, causes nausea, imbalance, inhibits the coordinated movements of the limbs, changes the proportion of components in the blood, causes anxiety and depression, and other symptoms of mental illnesses.

The pain threshold values are approximately 140 dB at approximately 40 Hz and 160 dB at approximately 3 Hz, higher levels can damage the inner ear. The main effect of prolonged exposure to high levels of noise is irreversible damage to the ciliary cells in Corti’s organ, which translates into a permanent increase in the hearing threshold (shift of the hearing threshold towards higher levels), which causes part of the speech signal, especially in the higher frequency range that affects its ability to recognize words lies outside the area of audible sounds. Long-term exposure to noise at the level of 78–86 dB (A) may lead to changes in enzymes in the peripheral blood. Laboratory studies have confirmed the impact of noise on the psychophysical fitness of drivers, and many countries have adopted noise exposure limits as 85 dB (A) ± 5 dB (A). Likewise, there is a risk when exposed to impulse noise. 

## 3. Noise Survey Objectives and Sources

In the case of occupational medicine in the field of hearing testing, it is based on experimental, repeatedly repeated measurements, and the assessment of the results with a varying degree of detail. It should be noted that some researchers are also involved in modeling acoustic phenomena [26]. In the shipping industry, on the ships, the main noise sources are machinery spaces. However, the floating storage and offloading vessels (FSOs) are stationary and most of the times stay at anchorage without running engine, thrusters, and propellers [12,16,27]. In such a case we can say that on FSOs the main noise sources are from the electrical generators and from the discharge of compressed air at the blast nozzles. For the operator, the next major source on FSO is the feed air inside the protective helmet, noise from abrasive blasting machinery, and noise from helicopter operation. Small blast cabinets as used by many workplaces in the metal industry are also significant sources of noise exposure for operators. Other sources of noise include machinery spaces, air compressors, ventilation systems, and air releases during pot blowdown.

In this paper we are going to describe a typical procedure connected to noise awareness on FSOs taking into consideration the best practices observed on FSO located in UK sector on North Sea [4] and FSO located offshore Australia [5]. Both FSOs are under selected Company management system [6], following same safety procedure and in most cases also using the same standard personal protective equipment (PPE) and same machinery on board. On both FSOs ships staff monitor noise exposure by recording noise in the daily/monthly noise log to identify and, where possible, correct high noise trends. In some cases, if needed, the occupational noise survey can be carried out by external company (e.g., occupational noise survey undertakes on 30 years old FSOs in 2016 by SVT Engineering Consultants [4,5]). In all our cases surveys were conducted to prevent health impact and hearing damage at plan approval, sea trails, and after major modifications. During the survey noise levels from processes and equipment were measured. The major objectives of the noise survey were to:

- Identify equipment and operations which have the potential to cause exposure standard to be exceeded. The exposure standard as specified in Control of Noise at Work Regulations (2005) based on European Union Directive 2003/10/EC (2003) and NOPSEMA Regulations (2006) are as follow:
- (a) 85 dB (A) averaged over an 8-h period for noise exposure (L_Aeq_,8h), or
- (b) 140 dB (C) for peak noise (L_Cpeak_).
- Designate areas where the average sound pressures level (L_Aeq_) exceeds 85 dB (A) or the peak noise level exceeds 140 dB (C).
- Evaluate noise exposures so that personnel exposed to noise levels above 85 dB (A) (L_Aeq_,_8h_) can be identified.
- Assess the adequacy of the personal hearing protectors already in use and of alternative protectors if required.
- Investigate noise sources and areas that contribute most to personnel noise exposure to determine the potential noise control options.

In collecting data for this report the following methodology was undertaken:

- Measure the average noise levels (L_Aeq_,_T_) at operator positions for noisy processes in order to establish noise exposures;
- Identify noisy equipment and processes and indicate the extent of high noise areas surrounding noisy equipment and processes;
- Measure noise contours throughout the process areas;
- Conduct interviews with personnel to attain work schedule information, for evaluation of noise exposures;
- Measure octave band noise levels for noisy equipment and processes, for assessment of hearing protectors for adequacy of protection;
- Conduct a hearing protection audit that covered available hearing protection, the use of hearing protection, and hearing protection signage location and visibility;
- Inspect dominant noisy equipment with consideration for noise control; and,
- Measure noise levels in accommodation cabins.

All measurements have been performed as required by Noise Management Plan (each FSO have its own ship-specific Noise Management Plan) with noise standards as per the National Code of Practice and Noise Control Policy (e.g., in Australian sector AS/NZS1269 (2005)).

With reference to survey carried out on example FSO by SVT Engineering Consultants sound level measurements were taken using a Bruel & Kjaer type 2270 sound level meter which meets the requirements for Type 1 sound level meters as specified in AS1259. The meter also meets the requirements for octave band filters as specified in IEC 1260 and AS/NZS 4476:1997.

The sound level meter was calibrated on site before and after measurements were taken, using a Bruel & Kjaer type 4231 reference sound source. As required by AS1269, noise exposure has been evaluated without considering the effects of any personal hearing protection. Where possible, measurements have been made when the prevailing conditions are most likely to produce typical noise emissions.

## 4. Audible Noise Measurements

**Figure 1:**
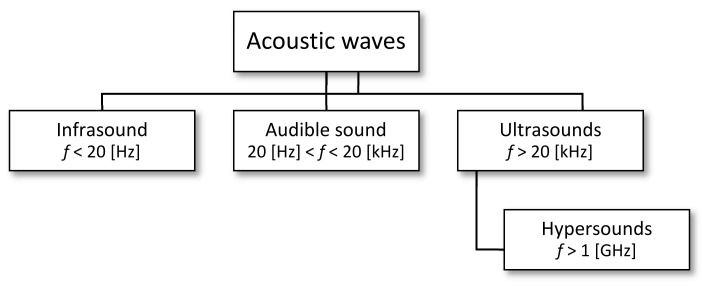
Types of noise.

Noise is defined as an undesirable, unpleasant or disruptive sound for a person at a given place and time. The types of acoustic waves in the frequency domain are shown in Figure 1.

**Figure 2:**
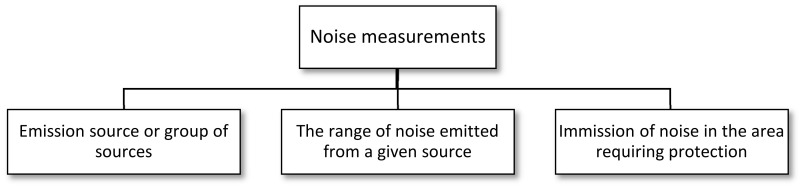
The need for noise measurements [28].

In the case of noise impact measurements, class 1 measuring devices are used. Measurements of the sound level are the first stage of the analysis. The advisability of conducting measurements is shown in Figure 2.

**Figure 3:**
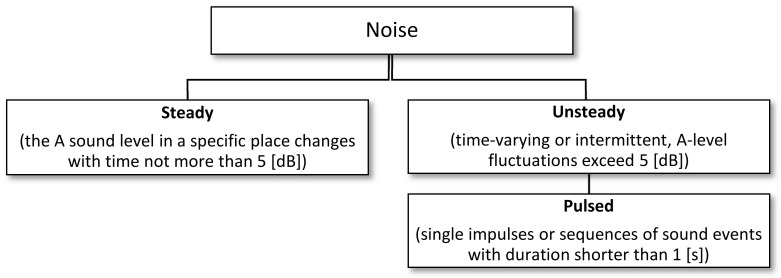
Classification of noise according to the time of duration [17].

For the steady noise, the A-weighted (L_Am_) or equivalent (L_Aeq_) sound level is determined for the assessment time. For transient noise, the A-weighted sound level (L_Aeq_) is determined for the above-mentioned time and the maximum A-sound level (L_Amax_), taking into account the slow time characteristics. The classification of noise due to level fluctuations during the exposure is shown in Figure 3.

The parameters to be assessed during the noise impact test are:

- Duration of sound events,
- Sound level (average, maximum, peak, equivalent),
- Exposure and level of exposure to noise.

The sound pressure level is the logarithm of the ratio of the sound pressure to the reference pressure:

(1) $$L_{p}=10\log\frac{p^{2}}{p_{0}^{2}}=20\log\frac{p}{p_{0}},dB$$

where:

*p*—measured value of the sound pressure in Pa,

*p_o_*—reference pressure, *p_o_* = 2 ×⋅10^−5^ Pa = 20 μPa = 0.00002 Pa.

The reference pressure po corresponds to the lower limit of audibility of the human ear, i.e., 0 dB. The sound pressure level measured at a given unit of time is called the instantaneous sound level and is often called SPL (Sound Pressure Level). The equivalent sound level Laeq characterizes a noise which varies with time. It expresses the same energy and at the same time the same risk of hearing damage as measured noise with varying levels. The equivalent level is given by the Equation (2) for group of sources or (3) for single source of noise [10,11]:

(2) $$L_{Aeq,T}=10\log\left[\frac{1}{T}\overset{n}{\underset{i=1}{\sum }}t_{i}\cdot 10^{0.1L_{Ai}}\right]$$

or

(3) $$L_{Aeq,T_{e}}=10\log\left[\frac{1}{T_{e}}\int_{0}^{T_{e}}{\left(\frac{p_{a\left(t\right)}}{p_{0}}\right)}^{2}dt\right]$$

where:

*L_Aeq.T_*—equivalent A-weighted sound level for the assessment time T,

*L_Ai_*—sound level operating at time ti,

*ti*—*L_Ai_* level sound duration,

*p_a_*—measured value of the sound pressure in Pa,

*T*, *T_e_*—noise exposition time.

Correction filters are used due to the properties of human hearing, consisting in the difference in the perception of sounds of the same level, but of different frequency; signal processing by correction filters with normalized characteristics (frequency weighting) is used.

**Figure 4:**
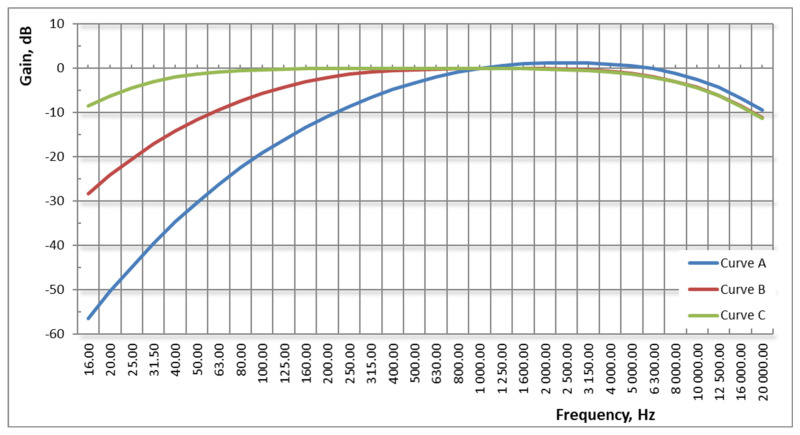
Correction filters characteristics.

The phenomenon of subjective perception of sounds—correction filters, see Figure 4:

- A—human ear reaction to sounds with low levels of 0–55 phons,
- B—human ear response to sounds with average levels of 55–85 phons,
- C—human ear response to high-level sounds over 85 phons.

## 5. Occupational Noise Survey

On all FSOs inside accommodation noise levels were measured in a sample of cabins and rooms. Measurements were taken approximately 1 m below the air condition vent (HVAC), and just above the pillow of the bed in accommodation cabins. Measurements were taken at a minimum length of 30 s, although a longer time was used where the noise was variable. Noise levels in cabins varied between 42 and 49 dB (A), exceeding e.g., the NOPSEMA guideline recommendation of 40 dB (A). These levels may cause a higher probability of sleep disturbance, which can result in increased fatigue, and potentially lead to an increase in accidents and/or injuries aboard the facility. However, on questioning personnel on board, there were no complaints with regards to cabin noise.

Each FSO has various workgroups that are divided into Similar Exposure Groups (SEG’s) e.g., watch-keepers, day workers, cadet/trainee, galley staff etc. The workforce is split into two crews which each do alternate roster of four weeks on, four weeks off (the numbers presented are for the entire workforce). Most personnel normally work on day shift (08:00 to 17:30), with the exception of watch-keepers, who work on a rolling 4 h on, 8 h off schedule.

Table 1 and Table 2 present the example of Similar Exposure Groups SEGs with the highest calculated noise exposure levels, exceeding the noise exposure standard of 85 dB (A) L_Aeq_,_8h_ recorded on FSO, based on SVT Engineering Consultants survey carried out in 2016 [5] and 2017 [4]. The calculations are based on average noise levels measured at places where employees are likely to be while performing their daily tasks during a working shift. The daily exposure level is an 8-h equivalent continuous A-weighted sound pressure level. Exposure calculations are estimates based on typical work patterns advised during interviews with representatives of each SEG.

After noise survey on each FSO, all the ship staffs were able to identify the noise hazards that most significantly contribute to the exposure of the entire workgroup. The Table 3 ranks the top 10 noise sources/activities on FSO according to the total workforce exposure. That is, the sum of the noise exposure across the entire workforce of each particular noise source or activity this workforce is exposed to. However, it should be also noted that the top 10 noise sources presented in Table 3 are not the highest noise levels measured during the survey, but the process, activity, or noise source that has the biggest influence on exposure of the workforce. For more information, see also Table 4 with overview of major noise sources on subjected FSOs, and on octave band measurements on Figure 7, with data collected based on noise surveys [4] and/or [5].

**Figure 5:**
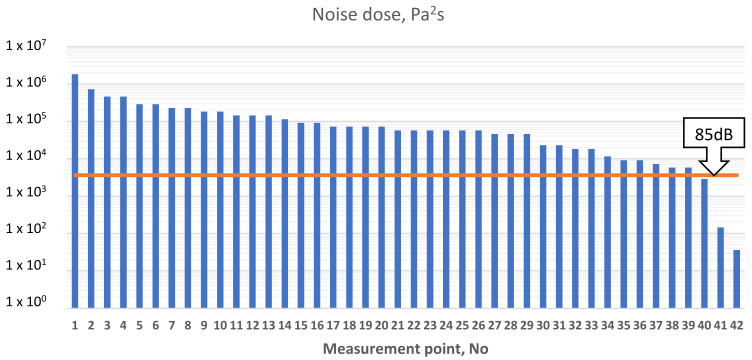
Daily exposure to noise E_A Te_ (noise dose).

**Figure 6:**
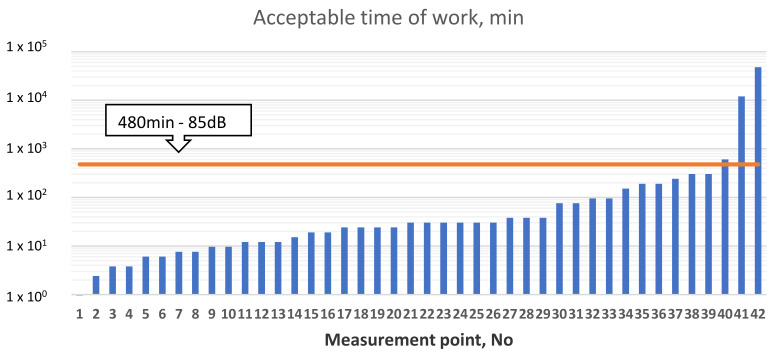
Acceptable time of work.

The equivalent of the noise exposure level, related to a work day is the value called the daily or weekly exposure to noise E_A Te_, so-called “noise dose” expressed in [Pa^2^s]. There is the following relationship between noise exposure and exposure level (4). The results for the points listed in the Table 4 are shown in Figure 5 and Figure 6.

E_A,Te_ = 1.15 × 10^−5^ × 10 ^0.1 Lex,8h^ (4)

**Figure 7:**
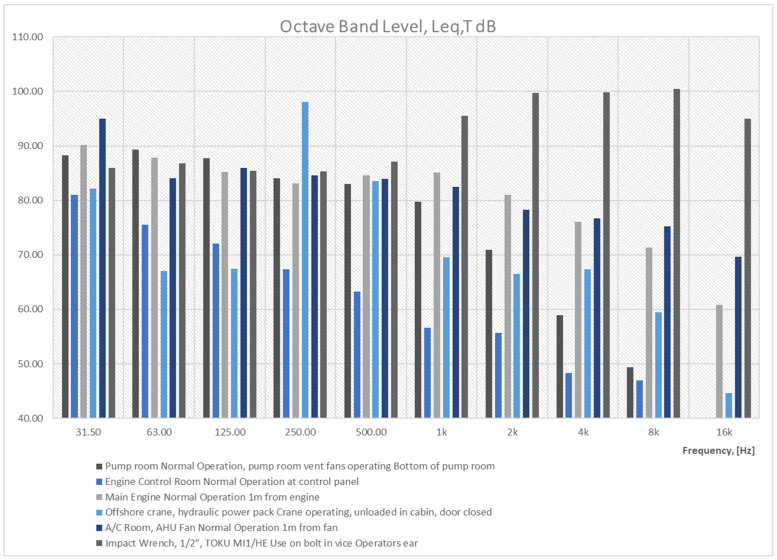
Overview of major noise sources on subjected FSO contributing to noise exposure for onboard workers in chosen areas, based on data survey from typical 1st generation FSO [1].

As it shown below, on Figure 6, work in these places without hearing protectors is not permitted. Hearing protectors are the easiest way to protect your hearing against the effects of noise. In this case, earmuffs and anti-noise earplugs can be used. Figure 7 shows an overview of major noise sources on subjected FSO contributing to noise exposure for onboard workers in chosen areas, based on octave band analysis.

For comparison, an indication of the level of noise experienced on subjected 1st generation FSO [4] in abrasive blasting processes was obtained from the following noise readings taken at operator ear level: air discharge from blast nozzle (112 to 119 dB (A)), feed air inside helmet (94 to 102 dB (A)), blast cabinets (90 to 101 dB (A)), air compressors (85 to 88 dB (A)). These were all above the prescribed level. Maximum noise levels up to 137 dB (A) and peak levels up to 145 dB (A) have been measured during blasting activities at the operator position when the abrasive runs out.

## 6. Discussion of Risk Assessment and Control Measures

As in general risk assessment in shipping industry, there is also a hierarchy of risk controls on FSO, which apply to noise and these are as follows:

- Isolation—This method can be used to isolate workers and other persons from noise exclusion zones and areas where noise levels are in excess of the exposure standard. All of these areas should be identified and entry restricted to persons with adequate hearing protection. Exclusion zones should be identified by appropriate signs in accordance with safety signs for the occupational environment which warn workers and others that high noise levels exist and that hearing protection is to be worn. A typical example on FSO installations is an engine control room, which separates the worker from the machinery.
- Elimination—at the design stage this may be done by substitution or purchasing of low noise equipment.
- Relocation or enclosing noisy equipment—e.g., blast cabinets, air compressors, and grit pots can be located in acoustic enclosures (sound proof) or separate rooms away from the work area so as not to expose other workers. In the open air, mobile enclosures, lined internally with sound absorbent material could be used at locations where noisy work has to be carried out and other people may be affected. Such enclosures could reduce operator exposure by about 5 to 20 dB (A) depending on construction. They could also reduce the exposures of people nearby.
- Substitute—use a quieter activity or process without vibroacoustic impacts, e.g., substituting an alternative surface preparation method. You should determine whether methods of cleaning other than abrasive blasting may be employed.
- Engineer out—use engineering control to reduce noise e.g., acoustic enclosure or noise haven for the worker, e.g., the noise from fans could be reduced using one or more of the following methods: replace the existing fans with aerodynamic, low noise types; implement variable speed drives on the fans, and adjust the fan speed depending on the ventilation requirements at the time (slower fan = less noise); install an in-duct noise silencer on the intake side of the fan and/or extend the ducts on the intake side of the fan to move the main noise source higher and further away from the deck.
- Administrative change—reduce exposure by changing the time of activity, e.g., when noise-producing equipment is stopped, reducing the number of personnel exposed, limit the length of exposure time.

## 7. Conclusions

Noise levels in shipping industry must meet industry and regulatory requirements. High noise areas must be clearly marked and personnel familiarization addresses this issue. Recreational facilities must be located as far removed from excessive noise as is practicable. Personnel working in or near high-noise level areas or performing work that generates high noise levels must wear approved hearing protection (earmuffs or earplugs). Such protection must have a sufficient NRR (noise reduction rating) to reduce noise exposure to permissible levels (80 dB). Hearing protection must be worn on all helicopter flights and workboat trips. In addition, other work activities must not contribute to further noise exposure. Unprotected workers and others close to the e.g., blasting process may also be exposed to excessive noise.

It must be also noted, that operators of small abrasive blasting cabinets on both FSOs were particularly at risk. However, they may not perceive the noise to be damaging because of the relatively short periods of use. However, average noise levels at the operator’s ears have been measured between 90 and 101 dB (A) on both FSOs (based on [4,5]). This means that at 101 dB (A), for instance, an exposure of unprotected ears of only 12 min is allowed in any 8-h shift so as not to exceed the exposure limit of 85 dB (A). In such cases when efforts have been made to mitigate noise, as far as reasonable and practical, evaluation should be undertaken to determine the success or reduction efforts. The successful strategy to reduce radiated noise should consider interactions and contributions from measures provided to achieve other objectives such as reduction of onboard noise and improvements in energy efficiency. When the analyzes were carried out, a whole range of international standards and regulations were also used that were not explicitly mentioned, but were written in Appendix A.

## Figures and Tables

**Table 1:** Calculated daily exposure level adjusted for 8 h (L_Aeq_,8h) prepared for each crew member (employee type in SEGs) on typical 1st generation (30 years old) floating storage and offloadings (FSOs) based on noise survey carried out in year 2016–2017 [4,5].

No.	Area/Employee Type	Number of Employees Exposed	Adjusted L_Aeq_,_8h_ dB(A)	No.	Area/Employee Type	Number of Employees Exposed	Adjusted L_Aeq_,_8h_ dB(A)
1.	Master, Chief Mate, 2nd Mate	6	80	7.	Chief Engineer	2	84
2.	3rd Mate	2	83	8.	1st Engineer	2	92
3.	Integrated Rating (watch-keeper)	6	80	9.	2nd Engineer	2	96
4.	Chief Integrated Rating	2	97	10.	3rd Engineer	2	92
5.	Integrated Rating (dayworker)	6	100	11.	Cadet/Trainee	6	97
6.	Chief Catering Attendant, Chief Cook	4	69	∑=		40	

**Table 2:** Similar exposure groups (SEGs) with a calculated adjusted daily exposure level (L_Aeq_,_8h_) > 85 dB(A) prepared based on data from noise survey carried out on typical 1st generation FSO in year 2016–2017 [4,5].

No.	SEG/Employee Type	Major Noise Sources Contributing to Exposure	Number of Employees Exposed	Adjusted L_Aeq_,_8h_ dB(A)	Sum of Group Exposure (Pa^2^hrs)
1.	Integrated Rating (dayworker)	Air chisel, needle guns	6	99.9	186.89
2.	Cadet/Trainee	Impact wrenches, needle guns, engine room	6	96.5	86.55
3.	Chief Integr.Rating	Air chisel, needle guns	2	97.3	34.01
4.	2nd Engineer	Diesel generators, engine room	2	96.2	26.80
5.	1st Engineer	Diesel generators, engine room	2	92.4	11.04
6.	3rd Engineer	Diesel generators, engine room	2	91.6	9.24

**Table 3:** Noise source risk ranking (from 1 to 10) taking into consideration typical work activity, noise sources, measured nose level, number of employees exposed, and sum of workforce exposure [4,5].

No.	Work Activity/Noise Source/Location	Measured Noise Level L_Aeq_,_t_ dB(A)	Number of Employees Exposed	Sum of Workforce Exposure (Pa^2^hrs)
1.	Air Chisels	112	8	116.7
2.	Needle-guns	102	14	67.2
3.	Impact Wrenches	106	14	65.5
4.	Engine room (boiler operation)	96	12	36.9
5.	Engine room (normal operation)	90	24	24.7
6.	Diesel Generators	99	2	18.2
7.	Engine Room Supply Fans (on deck)	88–100	22	12.1
8.	Offshore crane	92	8	4.9
9.	Angle grinders	93	14	4.1
10.	Winch hydraulic power packs	97	8	3.3

**Table 4:** Overview of major noise sources on subjected FSO contributing to noise exposure for onboard workers prepared based on data survey from typical 1st generation (30 years old) FSO [4].

No.	Noise Source Type & Make/Model	Operation/Activity	Measurement Position	L_Aeq_,_T_ dB(A)	L_CPeak_ dB(C)
1.	Air Chisel, TOKU AA-1.3F	Removing rusty valve flange bolt	Operators ear	112	130
2.	Emergency Generator	Normal operation	1 m from engine	108	125
3.	Impact Wrench, 1/2”, TOKU MI1/HE	Tightening valve flange bolt	Operators ear	106	130
4.	Needle-gun, Jet-chisel, JEX-24	Cleaning valve flange	Operators ear	106	125
5.	Boiler No.2 FD Fan	Boilers Operating	1 m from fan motor	104	122
6.	Pneumatic air buffer, wire wheel, Myton MAG W-40	Polishing steel	Operators ear	104	117
7.	Boiler No.1 FD Fan	Boilers Operating	1 m from fan motor	103	124
8.	Impact Wrench, 1/2”, TOKU MI1/HE	Use on bolt in vice	Operators ear	103	122
9.	Impact Wrench, 3/4”, JBS	Use on bolt in vice	Operators ear	102	117
10.	Needle-gun, Atlas Copco	Cleaning valve flange	Operators ear	102	116
11.	Ball pein hammer	Punching gasket in hole punch	Operators ear	101	132
12.	Drop saw, Hitachi CC14SF	Cutting steel angle	Operators ear	101	121
13.	Needle-gun, Jet-chisel, JEX-24	Working on main deck	Operators ear	101	119
14.	C Deck, Engine Room Supply Fans, STBD-Aft	Normal Operation	1 m from fan intake	100	121
15.	C Deck, Engine Room Supply Fans, Port-Aft	Normal Operation	1 m from fan intake	99	120
16.	Diesel Generator No.1	Normal Operation	1 m from engine	99	114
17.	C Deck, Engine Room Supply Fans, STBD-FWD	Normal Operation	1 m from fan intake	98	119
18.	A Deck, Pump Room Extract Fan	Normal Operation	1 m from fan discharge	98	118
19.	Angle grinder, 4”, Hitachi G10SD2	Grinding grid mesh	Operators ear	98	115
20.	Needle-gun, Atlas Copco	Working on main deck	Operators ear	98	112
21.	Steel mallet	Hitting impact spanner	Operators ear	97	130
22.	Steel mallet	Punching gasket on block	Operators ear	97	127
23.	C Deck, Engine Room Supply Fans, Port-FWD	Normal Operation	1 m from fan intake	97	120
24.	Emergency Generator	Normal Operation	1 m from open door, outside	97	115
25.	Bosun Store, FWD winch hydraulic power-packs	Power-packs operating	Between power packs	97	114
26.	Angle grinder, 9”, Bosch GWS 26-230H	Grinding grid-mesh	Operators ear	97	111
27.	Engine room average (boiler operation)	Boilers Operating	Average over 3 levels	96	121
28.	Upper Deck, Winch, STBD-Aft	Operating winch, unloaded	Operators ear	96	115
29.	Circular saw, Saw cat 3057-40, 7 1/4”	Free running	Operators ear	96	108
30.	Angle grinder, 5”, Makita 9565C	Grinding grid-mesh	Operators ear	93	110
31.	Offshore crane, hydraulic power pack	Crane operating, unloaded	Outside crane power pack hatch	93	108
32.	A/C Chiller Compressor	Normal Operation	1 m from compressor	92	107
33.	Offshore crane, hydraulic power pack	Crane operating, unloaded	In cabin, door closed	92	104
34.	Engine room average (normal operation)	Normal Operation	Average over 3 levels	90	113
35.	Main Engine	Normal Operation	1 m from engine	89	108
36.	Boiler Gas Piping	Normal Operation	1 m from piping	89	106
37.	Upper Deck, Engine Room Supply Fans	Normal Operation	‘Burma Road’, adjacent to A/C vent	88	111
38.	A/C Room, AHU Fan	Normal Operation	1 m from fan	87	107
39.	Upper Deck, Steering Gear Room Supply Fan	Normal Operation	1 m from fan intake	87	106
40.	Pump room, pump room vent fans operating	Normal Operation, vent fans operating	Bottom of pump room	84	106
41.	Exhaust fan, HVAC	General levels in Galley	Average in Galley	71	98
42.	Engine Control Room	Normal Operation	At control panel	65	93

## Data Availability

Given in the bibliography.

## Appendix A. Standards and Other Documentation Related to the Analysis of Results and Used in Research, and Not Indicated Directly in the Content

[S1] Australian and New Zealand Standards:

AS NZS 476 Acoustics, Octave Band and Fractional Octave Band Filters (2006/2016).
AS NZS 1270 Acoustics, Hearing protectors (1999/2002).
NZS 1269 Occupational noise management, Measurement and Assessment of Noise Emission and
Exposure (2005/2014).

[S2]Australian Standards:

AS 1259 Acoustics, Sound Level Meters and IEC60942 Electroacoustic, Sound Calibrators (1982).
AS1269, Workplace Health and Safety Queensland. Workers, Employers and Noise at Work (brochure) AS
AS 1319, Safety Signs for the Occupational Environment (1994)
AS 2254, Acoustics, Recommended Noise Levels for Various Areas of Occupancy in Vessels and Offshore
Mobile Platforms (1998).
AS 2533 Acoustics, Preferred Frequencies and Band Centre Frequencies (2002).

[S3] Declaration of Maritime Labour Compliance – Part II, RF1086 v.1 DMLC Part 2, Document accessible April 2018, (2018).

[S4] IMO (International Maritime Organization) resolution MEPC.1/Circ.833, Guidelines for the reduction of underwater noise from commercial shipping to address adverse impacts on marine life. (2014).

[S5] National Occupational Health and Safety Commission: National Code of Practice for Noise Management and Protection of Hearing at Work NOHSC: 2009 (2004) and NOHSC: 1007(2000).

[S6] National Occupational Health and Safety Commission: National Standard for Occupational Noise:

[S7] National Offshore Petroleum Safety and Environmental Management Authority (NOPSEMA)

Guideline—Occupational Health and Safety Noise Exposure Standard, (2014).

[S8] The Control of Noise at Work Regulation (2005), HSE Document ISBN 0-7176-6164-4, available on: http://www.hse.gov.uk/noise/regulations.htm (accessed on 12 November 2019) (2005/2008).

[S9] Work Health and Safety Regulation 2011, Noise and vibration, website: https://www.worksafe.qld.gov.au (accessed on 12 November 2019), Website accessed in April 2018.
